# Longer-term response to SARS-CoV-2 vaccination after allogeneic blood stem cell transplantation

**DOI:** 10.1007/s00432-026-06632-4

**Published:** 2026-10-01

**Authors:** Carl C. Crodel, Laura Gork, Wibke Göpel, Paul Linke, Konrad Sinn, Jenny Miethke, Andreas Hochhaus, Inken Hilgendorf

**Affiliations:** Innere Medizin 2, Hämatologie Und Onkologie, Universitätsklinikum Jena, Am Klinikum 1, 07747 Jena, Germany

**Keywords:** Allogeneic transplantation,, Infections, Vaccination, SARS-COV-2

## Abstract

**Introduction:**

Recipients of allogeneic haematopoietic stem cell transplantation (alloHSCT) are at increased risk of COVID-19 and lower vaccine immunogenicity. This study assessed vaccine immunogenicity and safety in these patients.

**Methods:**

We analyzed the serological response after the 2nd and 3rd SARS-CoV-2 vaccination as well as potential factors influencing this response. Data were obtained from internal hospital records. In addition, a questionnaire was used to evaluate the vaccination status, vaccination side effects and the course of expired COVID-19 disease.

**Results:**

In total, 114 patients (median age 62, range 27 – 70) were enrolled between July and September 2022, with 55 females (48.2%). One year later a follow up was conducted. Most patients (89.4%) were vaccinated at least twice, 66.7% received three vaccinations, and 10.5% refused the vaccination. The mRNA vaccines were predominantly used. Most patients responded and reached SARS-CoV-2 spike IgG titers after the 2nd (81.8%) or 3rd (96.1%) vaccination. Patients vaccinated within 24 months of alloHSCT had significantly lower anti-spike IgG levels than those vaccinated later (*p*=0.017). Common side effects included arm pain (34%), swelling (14%), and limb pain (17%).

**Conclusion:**

Vaccination was feasible and well-tolerated in alloHSCT recipients. Timing of vaccination influenced immune response, with a stronger response after the 3rd dose compared to the initial series.

**Supplementary Information:**

The online version contains supplementary material available at https://doi.org/10.1007/s00432-026-06632-4.

## Introduction

The coronavirus disease 2019 (COVID-19), declared a pandemic in March 2020 led to far-reaching restrictions on everyday life worldwide (Sharma et al. 2021; Mushtaq et al. 2021). People with immunodeficiency are at increased risk of infection, morbidity, mortality and delayed viral clearence, from severe acute respiratory syndrome coronavirus 2 (SARS-CoV-2), which is associated with a severe acute respiratory syndrome (Healy et al. 2022; Sharma, et al. 2021).

At the beginning of the pandemic, oncological patients have been reported to obtain at least a twofold higher risk of severe COVID-19 outcomes, including intensive care, invasive ventilation, and death (Yang et al. 2020; Liang et al. 2020). In recipients of allogeneic haematopoietic stem cell transplantation (alloHSCT), 15% developed severe COVID-19 in the period from March to August 2020 requiring ventilation, with a 30-day survival of 68% (Sharma, et al. 2021).

A German multicenter study of 130 alloHSCT recipients identified advanced age (> 60 years), active disease, calcineurin inhibitor therapy, and SARS-CoV-2 infection within the first post-transplant year as risk factors for increased mortality, with an overall mortality of 16.2% and a median time to death of 32 days (Schaffrath et al. 2022). Consequently, high-risk groups were prioritized for vaccination (Leclerc and Maury 2021).

However immunocompromised patients were mostly excluded from phase 3 trials evaluating SARSCoV-2 vaccines (Polack et al. 2020). Since other vaccines, e.g. annual influenza vaccination, leads to a reduced immune response in alloHSCT recipients compared to healthy individuals, as it has been shown by Avetisyan et al. for the humoral immune response (Avetisyan et al. 2008), a review of the effectiveness of the SARS-CoV-2 vaccines for this patient group is indispensable (Ljungman 2021). Patients after alloHSCT showed a reduced antispike IgG antibody response to SARS-CoV-2 vaccination compared to healthy individuals (Lindemann et al. 2021; Watanabe et al. 2022). Both the immunosuppressive treatment of patients after alloHSCT and an expired or existing graft-versus-host disease (GvHD) was also included in the suspicion of attenuating immune response to vaccines (Daudt et al. 2022).

Thus, identifying factors affecting vaccine response and evaluating the long-term development of SARS-CoV-2 antibody levels in alloHSCT recipients is essential to optimize vaccination strategies. The study aimed to evaluate the serological response and safety of SARS-CoV-2 vaccines after alloHSCT, assess vaccination status and serological responses, and identify factors with effect on vaccine response.

## Patients and methods

### Recruitment of participants

The study was approved by the institutional review board (Reg. Nr.: 2021–2421-Bef) and conducted in accordance with the Declaration of Helsinki. Data were collected after informed consent. Patients without written consent or insufficient German language skills were excluded. Eligible patients were identified from the clinic database and had received alloHSCT between 2012 and 2021 at the University Hospital of Jena (UKJ), with regular follow-up care. The recruitment of the alloHSCT recipients was done exclusively via our stem cell transplant outpatient clinic. Any surviving patients were contacted, without other specific selection criteria. Patient characteristics and vaccination status were collected via self-report questionnaires sent by mail, including vaccination dates (see supplement), vaccine types, and side effects, with clinical data obtained from electronic health records. Recruitment was performed between July and September 2022. Vaccination and status of infection were reassessed during follow-up visits from August to December 2023, based on the enrollment date. A multiple response set with the option of providing additional written information was used to enquire about the reasons for refusing vaccination.

### Laboratory analysis

Vaccine response was assessed by serological testing. Humoral responses were assessed using DiaSorin LIAISON® SARS-CoV-2 TrimericS IgG test system (Dietzenbach, Germany) measuring anti-spike and anti-nucleocapsid antibodies in the beginning. From 16 September 2021 on the DiaSorin CLIA test system was used, in accordance with the recommendations of the WHO. Results were standardized to BAU/ml according to WHO recommendations, and the combination of negative response in the questionnaire and negative anti-N antibodies were used to exclude prior asymptomatic infection, in the knowledge of a bias that not every previous infection can be ruled out. Serological non-response was defined as anti-spike IgG < 33.8 BAU/ml, as this is an assay cut-off and does not correlate with clinical outcomes.

### Analysis and statistics

Factors analyzed included age, sex, vaccine type, time from transplantation to vaccination, GvHD, and rituximab-induced B-cell depletion. Furthermore, the antibody response after a third (booster) vaccination was examined. Statistical analyses were performed using IBM^©^ SPSS^©^ Statistics version 28.0.1.1. A *p*-value < 0.05 was considered statistically significant. The Mann–Whitney U test was used for unpaired metric variables. This test was also used for unpaired ordinal variables. The Wilcoxon test was used for paired, non-normally distributed metric variables. The Spearman correlation was used as a linear model. Nominal variables were evaluated using the Chi-Square test.

## Results

### Patient characteristics

Of 185 contacted patients, 117 (63.2%) responded; for 6 patients (3.2%) lacked forwarding address. Three patients (2.6%) vaccinated before alloHSCT were excluded. In total, 114 recipients (female n=55; 48.2%) with a median age of 62 (range 27–70) years participated. Patient and transplant characteristics are summarized in Table 1. Follow-up visits were performed by 79.8% (91/114).

**Table 1:** Patient characteristics

		Frequency (n)	Percentage (%)
Diagnosis	Acute Leukemia	65	57.0
	Lymphoproliferative diseases	13	11.4
	Myelodysplastic neoplasia	18	15.8
	Myeloproliferative neoplasia	10	8.8
	Other diseases (myeloma, aplastic anemia)	8	7.0
Intensity of the conditioning	myeloablative	26	22.8
	reduced- intensity conditioning	88	77.2
Donor	related	10	8.8
	unrelated	104	91.2
Gender of donor	female	53	46.5
	male	61	53.5
HLA-Match	5/10	2	1.8
	9/10	20	17.5
	10/10	92	80.7
Medication for GvHD prophylaxis	Calcineurin inhibitors	112	98.2
	Antithymocyte globulin	101	88.6
	Methotrexate	82	71.9
	Mycophenolate mofetil	29	25.4
Treatment of Epstein Barr virus reactivation	Rituximab	53	46.5

The median time from alloHSCT to calcineurin-inhibitor (CNI) cessation was 180 days (range 30–3226 d). Median interval to last rituximab administration was 60 days (range 2–2379 d). Acute GvHD occurred in 36.8% (n = 42/114) of patients at any time since transplant, with 40.5% (n = 17/42) requiring systemic treatment. Overall, 40.5% (n = 17/42) of patients with acute GvHD also developed chronic GvHD. In total, 28.9% (n = 33/114) developed chronic GvHD. Severity grades are depicted in Table 2.

**Table 2:** Severity of GvHD

Type of GvHD	Severity level	Frequency (n)	Percentage (%)
Acute GvHD n =42	°I	28	66.7
	°II	10	23.8
	°III	4	9.5
	°IV	0	0
Chronic GvHD	mild	24	72.7
	moderate	6	18.2
	severe	3	9.1

At second vaccination date 32 patients developed any kind of GvHD. 21.9% (n = 7/32) of them were still receiving immunosuppressants for the treatment of GvHD. The drugs used were prednisolone (57.1%; n = 4/7), budesonide (14.3%; n = 1/7), or CNI (28.6%; n = 2/7). The use of these immunosuppressants had no significant effect on the vaccine response (p = 0.714). Anti-spike IgG antibody production did not differ significantly between patients who developed GvHD and those who showed no signs of GvHD after allogeneic HSCT (*p* = 0.653).

At third vaccination date immunosuppressive treatment for GvHD was required in 14.3% (n = 3/21) of the patients. The immunosuppressive medications used were prednisolone (66.7%; n = 2/3) or budesonide (33.3%; n = 1/3). The use of immunosuppressants at the time of the third vaccination did not significantly influence the vaccine response (*p* = 0.841).

### SARS-CoV-2 vaccination status

**Fig. 1:**
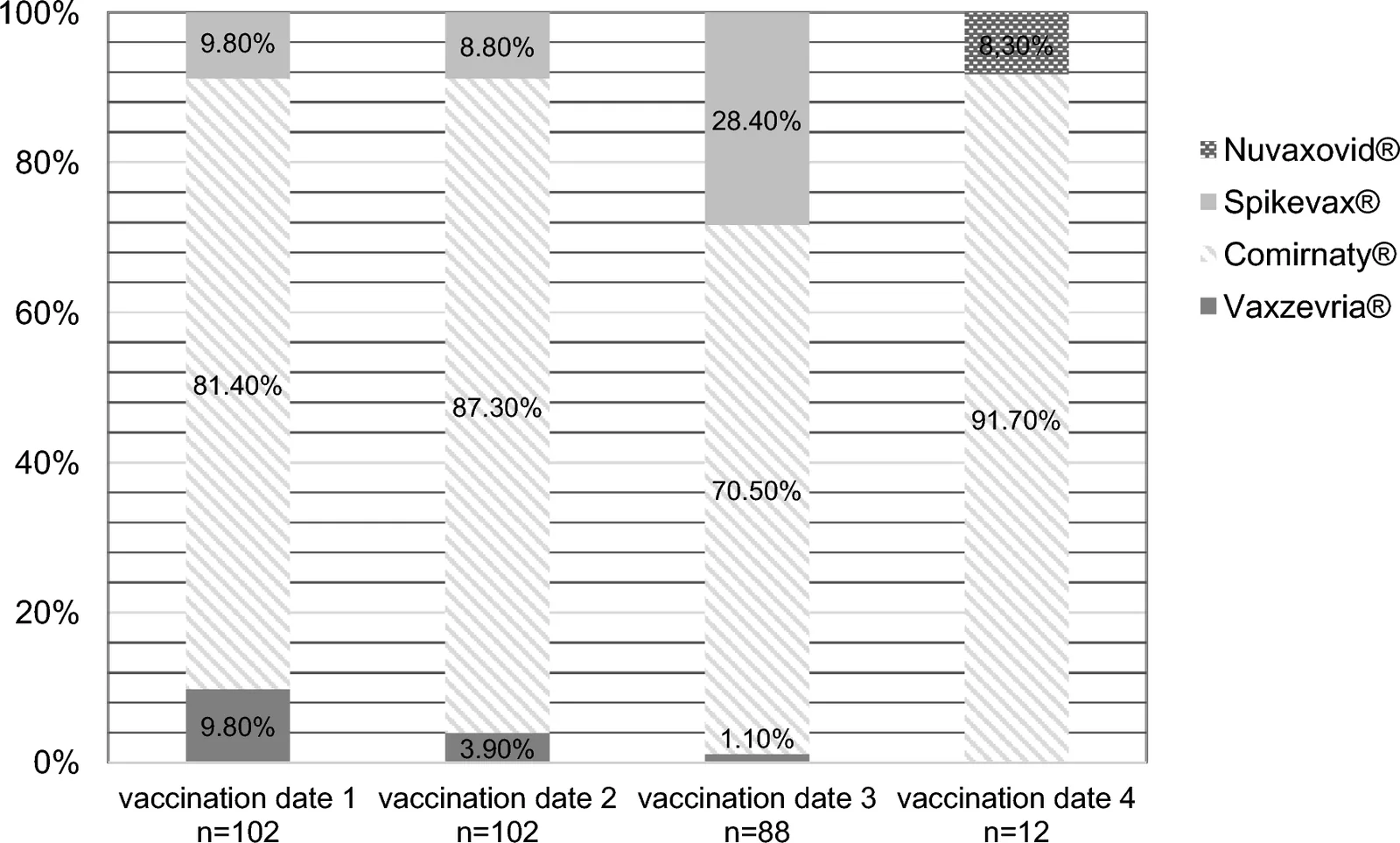
Administered vaccinations by the different timepoints. Used vaccines were the mRNA-based vaccines Comirnaty® from Biontech/Pfizer and Spikevax® from Moderna and the protein-based vaccine Nuvaxovid® from Novavax and Vaxzevria® from AstraZeneca

Overall, 89.5% (n = 102/114) of alloHSCT recipients received at least two SARS-CoV-2 vaccinations. The median time from alloHSCT to first vaccination was 1147 days (range 139–2978 d), with median intervals of 31 days (range 21–387 d) between the first and second vaccine, 184 days (range 43–307 d) between the second and third, and 146 days (range 91–356 d) between the third and fourth, respectively. The majority of patients (74.5%, 76/102) received three vaccinations. The mRNA-based vaccines Comirnaty® from Biontech/Pfizer (vaccination date = VD; VD 1: n=82/102; VD 2: n=98/102; VD3: n=62/88) and Spikevax® from Moderna (VD1: n=10/102; VD2: n=9/102, VD3: n= 25/88, the protein-based vaccine Nuvaxovid® from Novavax (VD4: n=1/12), and Vaxzevria® from AstraZeneca (VD1: n=10/102; VD2: n= 4/102; VD3: n=1/88) were used. Comirnaty® were used most frequently (Fig. 1).

Among the study population 12 patients were unvaccinated due to medical concerns (25%, n = 3/12), no willingness to be vaccinated (25%, n = 3/12) or other reasons (16.7% n = 2/12) like fear of possible side effects, secondary diseases caused by the vaccines and doubts regarding the sufficiently tested efficacy of the vaccines. Two patients did not answer the question (16.7%).

### COVID-19

Overall, 38.6% (n = 44/114) of alloHSCT recipients reported at least one episode of COVID-19, with most cases being mild (90.9%;n = 40/44) or moderate (9.1%; n = 4/44) based on the classification of Cordonnier and colleagues (Cordonnier et al. 2006). At time of illness, 38.6% (n = 17/44) were unvaccinated. In this group, 82.4% (n = 14/17) reported a mild and 17.6% (n = 3/17) a moderate course of COVID-19. After at least one administered SARS-CoV-2 immunization, 61.4% (n = 27/44) of patients suffered from COVID-19. Of these, 96.3% (n = 26/27 described the self reported disease severity as a mild course and one person (3.7%) reported about moderate symptoms. Disease severity did not differ significantly between vaccinated and unvaccinated patients (*p* = 0.117) (Table 3 and 4).

**Table 3:** Comparison of the frequency of vaccine side effects

Vaccination	Vaccines	At least one side effect		No side effects	
		Frequency (n)	Percentage (%)	Frequency (n)	Percentage (%)
First vaccination	Vaxzevria® n=10	6	60.0	4	40.0
	Comirnaty® n=83	35	42.2	48	57.8
	Spikevax® n=9	6	66.7	3	33.3
	Total n=102	47	46.1	55	53.9
Second vaccination	Vaxzevria® n=4	0	0	4	100
	Comirnaty® n=89	38	42.7	51	57.3
	Spikevax® n=9	3	33.3	6	66.7
	**Total n=102**	41	40.2	61	59.8
Third vaccination	Vaxzevria® n=1	0	0	1	100
	Comirnaty® n=62	23	37.1	39	62.9
	Spikevax® n=25	9	36.0	16	64.0
	**Total n=88**	32	36.4	56	63.6
Fourth vaccination	Comirnaty® n=11	4	36.4	7	63.6
	Nuvaxovid® n=1	0	0	1	100
	**Total n=12**	4	33.3	8	66.7

**Table 4:** Side effects of vaccination

Side effects	First vaccination n=102	Second vaccination n=102	Third vaccination n=88	Fourth vaccination n=12
Headaches	9 (8.8%)	9 (8.8%)	11 (12.5%)	/
Limb pain	17 (16.7%)	15 (14.7%)	9 (10.2%)	2 (16.7%)
Fever	7 (6.9%)	5 (4.9%)	2 (2.3%)	1 (8.3%)
Shivering	8 (7.8%)	5 (4.9%)	5 (5.7%)	1 (8.3%)
Fatigue/Tiredness	4 (3.9%)	8 (7.8%)	4 (4.5%)	/
Pain in the arm	35 (34.3%)	33 (32.4%)	21 (23.9%)	3(25.0%)
Reddening/ swelling of the arm	14 (13.7%)	13 (12.7%)	9 (10.2%)	1 (8.3%)
New onset/ reactivated GvHD	2 (2.0%)	2 (2.0%)	/	/

### Factors with effect on vaccination response

For the second vaccination, the vaccines Vaxzevria® (1.5%; n = 1/66), Spikevax® (6.1%; n = 4/66) and Comirnaty® (92.4%; n = 61/66) were used. Antibody responses differed significantly between vaccines (Kruskal–Wallis test, *p* = 0.016) and were higher with Spikevax® than Comirnaty® (*p* = 0.008). However, with the caveat that the number of patients is significantly lower for Spikevax®. After administration of Comirnaty® for second vaccination, 19.7% (n = 12/61) fell below this threshold. Half of these patients with serological non-response (50.0%; n = 6/12), suffered from GvHD, with 33.3% (n = 2/6) of patients on immunosuppressants at time of second vaccination. The period between alloHSCT and administration of second vaccine dose was less than 6 months in 25.0% (n = 3/12) and less than 12 months in 41.7% (n = 5/12). No vaccine failure was observed in those who received Spikevax®.

At data collection, 40.9% (n = 27/66) of patients were younger than 60 years. Anti-spike IgG levels were significantly lower in patients aged ≥ 60 years compared with those < 60 years (*p* = 0.008) (Table 5).

**Table 5:** Vaccination response by different factors

			Median (BAU/ml)	Mean (BAU/ml)	Standard deviation (BAU/ml)	Range (BAU/ml)	*p*-value
Influence of vaccination schedule	Two times Spikevax®	n = 4/66	4470.0	5320.65	4848.26	1040.0–11300.0	***p* = 0.008**
	Two times Comirnaty®	n = 61/66	673.4	1284.73	3848.66	< 4.8–29,700.0	
	Three times Spikevax®	n = 11/43	8120.0	8965.46	4451.75	305.0–15600	***p* = 0.029**
	Three times Comirnaty®	N = 32/43	1800.0	5965	8038.72	< 4.8–36,100.0	
Influence of patient age after second vaccination	< 60 years	27/66	1040.0	2861.47	5977.17	< 4.8–29,700.0	***p* = 0.008**
	> 60 years	39/66	431.60	574.57	521.27	< 4.8–1730.0	
Influence of patient age after third vaccination	< 60 years	19/43	3425.0	6008.65	8246.18	6.8–36,100.0	*p* = 0.229
	> 60 years	24/43	8120.0	7648.53	6157.32	< 4.8–15,600.0	
Influence of sex (after second vaccination)	Female	33/66	1040.0	2374.15	5485.24	< 4.8–29,700	*p* = 0.063
	Male	33/66	656.0	646.09	574.85	< 4.8–2330.0	
Influence of sex (after third vaccination)	Female	21/43	5110.0	7201.98	5964.71	< 4.8–18,300.0	*p* = 0.319
	Male	22/43	2310.0	6285.83	8603.32	6.8–36,100.0	
Influence of the time interval between alloHSCT and the second vaccination	Second Vaccination less than 12 month after transplantation	15/66	338.0	3174.96	7865.69	< 4.8–29,700.0	*p* = 0.370
	Second Vaccination more than 12 month after transplantation	51/66	782.6	1020.46	14,331.0	< 4.8–7590.0	
	Second Vaccination less than 24 month after transplantation	30/64	332.5	1841.93	5643.34	< 4.8–29,700.0	***p* = 0.017**
	Second Vaccination more than 24 month after transplantation	34/64	888.3	1286.74	1661.49	< 9.88–7590.0	
Influence of calcineurin inhibitors (after second vaccination)	Discontinuation of CNI less than 6 months before 2nd vaccination	11/63	327.0	4021.49	9140.11	< 4.8–29,700.0	*p* = 0.221
	Discontinuation of CNI more than 6 months before 2nd vaccination	52/63	808.6	1042.33	1415.86	< 4.8–7590.0	
	Discontinuation of CNI less than 12 months before 2nd vaccination	22/63	332.5	2315.24	6556.07	< 4.8–29,700	*p* = 0.087
	Discontinuation of CNI more than 12 months before 2nd vaccination	41/63	834.6	1158.59	1552.90	9.88–7590.0	
Influence of B-Cell-Depletion with Rituximab (after second vaccination)	with Rituximab therapy (total)	33/66	656.0	848.74	1344.5	< 4.8–7590.0	*p* = 0.247
	without Rituximab therapy (total)	33/66	873.6	2171.49	5408.14	< 4.8–29,700.0	
	Second vaccination 6–12 month after Rituximab	9/33	67.06	273.72	386.46	< 4.8–> 1040.0	***p* = 0.014**
	Second vaccination > 12 month after Rituximab	24/33	799.5	1075.23	1506.93	< 4.8–7590.0	
Influence of GvHD	Second vaccination in patients with GvHD at any time	32/66	759.6	2321.44	5589.49	< 4.8–29,700.0	*p* = 0.653
	Second vaccination in patients without GvHD at any time	34/66	743.6	746.52	633.76	< 4.8–2430	

A p-value < 0.05 was considered statistically significant

After second (*p*=0.063) and third (*p* = 0.319) vaccination, women showed numerically higher, without reaching statistical significanceantibody responses than men, although this was not statistically significant. The Wilcoxon test was used to assess the booster effect of a third vaccination on anti-spike IgG levels. Among the 88 patients receiving at least three vaccinations after alloHSCT, 36.4% had serological data after both the second and third doses; all were anti-nucleocapsid IgG negative and did not report symptoms of COVID-19.

After the second vaccination, the median IgG antibody level was 653.0 (range < 4, 8–7590) BAU/ml and increased to 3550.0 (range < 4, 8–36,100) BAU/ml after third vaccination (*p* < 0.001).

As antibody data after the first dose were limited, the effect of the interval between alloHSCT and second vaccination was analyzed. Shorter intervals between alloHSCT and the administration of second vaccination influenced the response. In patients who received a second vaccination within 6 (n=5) or 12 (n=15) months after alloHSCT anti-spike IgG levels does not differ than in those who were vaccinated later (≤ 6 *p* = 0.194 and / ≤ 12 months: *p* = 0.370). In contrast, patients vaccinated more than two years after alloHSCT had significantly higher antibody responses than those vaccinated within two years after transplantation (*p* = 0.017).

Among SARS-CoV-2–naïve patients with antibody data after the second vaccination 50.0% (n = 33/66) had received rituximab due to EBV-reactivation. Anti-spike IgG responses were significantly lower when rituximab was given 6–12 months before vaccination compared with > 12 months (*p* = 0.014). The median time interval between last rituximab therapy and second vaccination was 555 days (range 105 – 3055 d) and 807 days (range 174 – 2471 d) until third vaccination, respectively. Antibody levels after second dose increased with longer intervals since rituximab (p < 0.001), whereas no association was observed after third vaccination (*p* = 0.978) (Table 5).

Anti-spike IgG responses did not differ significantly between patients with GvHD (n=42) or without, either after second vaccination (*p* = 0.653) or third vaccination (*p* = 0.395).

### Side effects of vaccination

More than one third of patients reported at least one side effect after the first (46.1%; n = 47/102), second (40.2%; n = 41/102) and third vaccination (36.4%; n = 32/88), with no significant differences between vaccines (V1 / V2 / V3: Chi-square *p*-value = 2.826 / 3.096 / 0.587) (Table 3).

Adverse events were mostly mild, including local reactions and transitory flu-like symptoms like fever, chills, headache or limb pain as well as tiredness or fatigue. Reactivation or new onset of GvHD occurred in 2.0% (n = 2/102) of patients after first or second vaccination, but not after subsequent doses (Table 4).

## Discussion

Due to the delayed immune reconstitution after alloHSCT, it is assumed that vaccine induced serological response rates in patients after a transplant are reduced compared to the general population (Karras et al. 2013, Dhédin et al. 2014). However, recipients of alloHSCT demonstrate stronger antibody responses to SARS-CoV-2 vaccination than those who have undergone solid organ transplantation (Yeshurun et al. 2022).

As no established anti-spike IgG cutoff for vaccine response exists (Voysey et al. 2021, Loubet et al. 2021), a threshold of 33.8 BAU/ml was applied. The SARS-CoV-2 antibody concentration was evaluated in 66 patients after second and in 43 after third vaccination. Most alloHSCT recipients showed a response, with anti-spike IgG levels > 33.8 BAU/ml in 81.8% after second and 96.1% after third vaccination.

The proportion of alloHSCT recipients with adequate vaccine responses in this study is comparable to previous reports, with seropositivity after two doses observed in about 72–81% of patients (Pabst et al. 2022, Meyer et al. 2022). In addition, a German bicentric study reported on 72.0% of patients with measurable antibodies against the spike antigen after the second vaccination with mRNA and vector-based vaccines (Hütter-Krönke et al. 2023). Consistent with other studies using mRNA vaccines, most alloHSCT patients developed detectable anti-spike antibodies after the second vaccination (Yeshurun et al. 2022; Shem-Tov et al. 2022; Ram et al. 2021; Piñana et al. 2022; Majcherek et al. 2022; Watanabe, et al. 2022; Jullien et al. 2022).

A third vaccination is intended to prevent waning immunity and improve protection against SARS-CoV-2 variants such as Delta and Omicron (Chevallier et al. 2022). In the general population, booster doses increase neutralizing antibodies against the wild-type virus and multiple variants (Falsey et al. 2022; Choi et al. 2021).

In our study, 32 SARS-CoV-2–naïve patients had serological data after both the second and third vaccinations, showing a significant increase in median antibody levels from 653.0 BAU/ml after the second vaccination to 3550.0 BAU/ml after the booster (*p* < 0.001). In contrast, other studies have reported weaker antibody responses in alloHSCT recipients after two doses of Comirnaty®. However, the administration of a third vaccination led to a strong increase in antibody production (Chevallier, et al. 2022; Redjoul et al. 2021; Albiol et al. 2022; Beerlage et al. 2022). Antibody responses were significantly higher after vaccination with Spikevax® than with Comirnaty® (Md = 4470,0 BAU/ml; range > 1040,0–11300,0 BAU/ml versus Md = 673,4 BAU/ml; range < 4,8–29,700,0 BAU/ml; *p* = 0.008), possibly due to higher mRNA content. Kaplonek et al. reported stronger epitope-specific responses after two doses of Spikevax®, including higher receptor-binding and N-terminal domain–specific IgA levels, as well as increased antibodies mediating neutrophil phagocytosis and natural killer cell activation, compared with Comirnaty® (Kaplonek et al. 2022). Patients younger than 60 tended to show stronger antibody responses after the second and third vaccinations than older patients (V2 p = 0.008 / V3 p = 0.229), although a general age-related correlation could not be confirmed (V2 p = 0.142 / V3 p = 0.483), likely due to small sample size (V2 n = 66 / V3 n = 43). In contrast, studies in healthy populations report reduced anti-spike IgG responses with increasing age (Li et al. 2022; Loubet et al. 2021), though the underlying mechanisms remain unclear (Li, et al. 2022).

Notably, women tended to develop higher anti-spike IgG antibody levels than men after the second and third vaccine dose (V2 p = 0.063 / V3 p = 0.319), however without statistical significance. Similar findings have been reported by other studies, showing stronger anti-spike IgG antibody responses in women than in male alloHSCT recipients after SARS-CoV-2 vaccination (Ram, et al. 2021; Lindemann, et al. 2021; Majcherek, et al. 2022; Kokogho et al. 2023).

Patients vaccinated within 12 months after alloHSCT tended to have lower anti-spike IgG levels than those vaccinated later (*p* = 0.370), without significance, likely due to small sample size. This aligns with previous findings showing reduced seropositivity when vaccination occurs within one year post-transplant (Piñana, et al. 2022). Accordingly, a longer interval between alloHSCT and vaccination—particularly ≥ 24 months—is associated with significantly higher antibody responses in our study (*p* = 0.017).

In this study, prior rituximab therapy was not independently associated with a significantly reduced antibody response (*p* = 0.247). However, longer intervals between the last rituximab treatment and vaccination were associated with higher anti-spike IgG levels (*p* < 0.001). Patients vaccinated 6–12 months after rituximab had significantly lower antibody responses than those vaccinated ≥ 12 months later after the second and third dose (V2 p = 0.014 / V3 p = 0.021). Reduced B-cell numbers likely explain the lower anti-spike IgG responses observed in patients vaccinated within one year after rituximab treatment. Restrictions are based in very small subgroups after second vaccination.

Although GvHD have been suspected to impair vaccine responses (Yeshurun, et al. 2022; Sengsayadeth et al. 2022), we observed no significant reduction in anti-spike IgG levels in patients with GvHD after the second or third vaccination (V2 p = 0.653 / V3 p = 0.395) in our cohort, nor an effect of ongoing immunosuppressive therapy (*p* = 0.714).

Several studies (Jullien, et al. 2022; Beerlage, et al. 2022)albeit with respective limitations in data collection, observed no negative impact of prior GvHD on vaccine response, whereas Yeshurun et al. (Yeshurun, et al. 2022) reported higher seronegativity in patients with current or past GvHD after two Comirnaty® doses. Evidence is mixed when acute and chronic GvHD are analyzed separately: acute GvHD and its treatment showed no effect in some studies (Ram, et al. 2021), while others reported reduced responses under treatment with steroid or CNI (Kokogho, et al. 2023). In contrast, according to Meyer et al. (Meyer et al. 2022), treatment with steroids and CNI at the time of vaccination decreases SARS-CoV-2 antibody production. Hütter-Krönke et al. (Hütter-Krönke, et al. 2023) established an association between a low antibody response rate and ongoing immunosuppressive therapy at the time of the second SARS-CoV-2 vaccination. Chronic GvHD generally showed no significant impact (Huang et al. 2022), though available data are limited by small and heterogeneous cohorts. In our study, we found no effect of GVHD or its treatment on the development of an immune response to vaccination.

The safety profile of SARS-CoV-2 vaccines in our cohort was favorable, with predominantly mild side effects such as local injection-site reactions and transient systemic symptoms, comparable to those of the general population (Ge et al. 2022). Consistent with previous studies, SARS-CoV-2 vaccination after alloHSCT is considered safe and feasible (Sengsayadeth et al. 2022; Li, et al. 2022; Piñana, et al. 2022; Matkowska-Kocjan et al. 2021; Majcherek, et al. 2022; Ali et al. 2021). Common vaccination reactions in alloHSCT recipients include injection-site pain and mild systemic symptoms such as fever, chills, and body aches (Canti et al. 2021; Watanabe, et al. 2022; Shem-Tov, et al. 2022; Ali, et al. 2021; Matkowska-Kocjan, et al. 2021).

Due to its observational design, this monocentric retrospective study has limitations, including a small sample size, lack of virus variant identification, and non-standardized vaccination and antibody testing intervals. Furthermore, cellular immune responses were not assessed, as well as lymphocyte counts or T cell/B cell counts pre- or post-vaccination. Since regular immunocytological examinations are not part of our standard routine. There is also a high rate of EBV reactivations with consecutive administration of rituximab, which has an impact on the vaccine response. Side effects were collected retrospectively by chart review, which can lead to a recall bias.

Nevertheless, this is, to our knowledge, one of the first surveys to evaluate vaccination status and antibody responses in HSCT recipients at multiple time points including a follow-up one year after first vaccination.

In summary, we demonstrate that SARS-CoV-2 vaccination after alloHSCT is feasible, safe, and induces protective antibody responses in a substantial proportion of patients.

## Supplementary Information

Below is the link to the electronic supplementary material.

Supplementary file1 (DOCX 469 kb)

## Data Availability

All data supporting the findings of this study are available within the paper and its Supplementary Information.
