# Supplementary material for: Longer-term response to SARS-CoV-2 vaccination after allogeneic blood stem cell transplantation
Source: J Cancer Res Clin Oncol. 2026 Oct 1;152(10):191. doi: 10.1007/s00432-026-06632-4 (PMC13634024; doi:10.1007/s00432-026-06632-4)
Supplement: Supplementary file 1 [file 432_2026_6632_MOESM1_ESM.docx]

**Long-term response to SARS-CoV-2 vaccination after allogeneic blood stem cell transplantation**

**Supplemental Data**

Contents

1 Supplemental tables

Supplemental table 1 - Anti-spike IgG antibodies after mRNA vaccine administration

Supplemental table 2 - SARS-CoV-2 antigen contact frequency

2 Supplemental figures

Supplemental figure 1 - Impact of a third booster vaccination on the anti-spike IgG

antibody response

Supplemental figure 2 - Anti-spike IgG antibody production depending on SARS-CoV-2

antigen contact frequency

Supplement figure 3 - Anti-spike IgG antibody response to the second vaccination with

increasing age

3 Questionaire

**1 Supplemental tables**

**Supplemental table 1 - Anti-spike IgG antibodies after mRNA vaccine administration**

|  | **Anti-spike IgG antibodies after V2**  **in BAU/ml** | | | **Anti-spike IgG antibodies after V3**  **in BAU/ml** | | |
| --- | --- | --- | --- | --- | --- | --- |
|  | **Total**  **N=72** | **Comirnaty®**  **n=66** | **Spikevax®**  **n=6** | **Total**  **N=51** | **Comirnaty®**  **n=37** | **Spikevax®**  **n=14** |
| Md | 848.9 | 743.6 | 4470.0 | 3490.0 | 1820.0 | 6740.0 |
| M | 3164.0 | 2058.8 | 15320.4 | 6796.6 | 6589.6 | 7343.6 |
| SD | 99884.1 | 6010.7 | 26909.9 | 8072.9 | 9005.6 | 5062.8 |
| Min | <4.8 | <4.8 | 1040.0 | <4.8 | <4.8 | 1370.0 |
| Max | 69600.0 | 35100.0 | 69600.0 | 36100.0 | 36100.0 | 15600.0 |
| IgG-AK  NkAg | n=5  6.9% | n=4  6.1% | n=1  16.7% | n=5  9.8% | n=3  8.1% | n=2  14.3% |

V2: SARS-CoV-2 second vaccination; V3: SARS-CoV-2 third vaccination; BAU/mL: binding antibody units per milliliter; Md: median; M: mean; SD: standard deviation;
Min: minimum; Max: maximum; IgG-Ak NkAg: immunoglobulin G antibodies against the nucleocapsid antigen.

**Supplemental table 2 - SARS-CoV-2 antigen contact frequency**

| **SARS-CoV-2**  **antigen contacts** | **Total**  **N=52** | **M**  **BAU/ml** | **Md**  **BAU/ml** | **Min**  **BAU/ml** | **Max**  **BAU/ml** | **SD**  **BAU/ml** |
| --- | --- | --- | --- | --- | --- | --- |
| **1** | 5 (7.8%) | 225.7 | 193.0 | <4.8 | 472.0 | 193.0 |
| **2** | 1 (1.6%) | / | / | / | / | / |
| **3** | 22 (34.4%) | 6021.0 | 1640.0 | 58.0 | 712000.0 | 14982.8 |
| **4** | 30 (46.8%) | 7721.4 | 4090.0 | <4.8 | 32000.0 | 9131.7 |
| **5** | 5 (7.8%) | 4648.6 | 3900.0 | 132.0 | 10300.0 | 4690.2 |
| **7** | 1 (1.6%) | / | / | / | / | / |

BAU/mL: binding antibody units per milliliter; Md: median; M: mean; SD: standard deviation;
Min: minimum; Max: maximum;

**2 Supplemental figures**

**Supplemental figure 1** - illustrates the positive influence of a third vaccination in the sense of a

booster on anti-spike IgG antibody production, BAU/ml: binding antibody units per millilitre

**Supplemental figure 2** - illustrates the anti-spike IgG antibody response resulting from varying numbers of SARS-CoV-2 antigen contacts, BAU/ml: binding antibody units per millilitre

**Supplemental figure 3** - outlines the influence of increasing age on the SARS-CoV-2

vaccine response, BAU/ml: binding antibody units per millilitre

UPN Nummer

Universitätsklinikum Jena · Klinik Innere Medizin II · Postfach · 07740 Jena

# Questionnaire: COVID-19 Infection and COVID-19 Vaccination after Stem Cell Transplantation

Date of transplantation:	|__|__|.|__|__|.|__|__|__|__|

Day	Month	Year

Sex:	1 female	2 male	3 diverse

Date of birth:	|__|__|.|__|__|.|__|__|__|__|

Day	Month	Year

**1. Have you had COVID-19?**	1 yes	2 no

If yes:

Number of infections	1	Once

2	Twice

3	Three times

4	More than 3 times

Last Covid-19-Infection	|__|__|.|__|__|.|__|__|__|__|

Day	Month	Year

Course of disease:	1 Mild (no to mild symptoms)

2 Moderate (hospital admission)

3 Severe (intensive care treatment)

**Did you receive antibody therapy for the treatment of**	1 yes

COVID-19 (e.g. Bamlanivimab/Etesevimab, Ronapreve®	2 no

(Casivirimab/Imdevimab), Xevudy® (Sotrovimab),	99 unknown

Evusheld® (Tixagevimab/Cligavimab))?

**2. Have you had to be treated for an infection other than**	1 yes

**COVID-19 within the last 6 months?**	2 no

If yes:

Type of treatment	1 Single outpatient

2 Single inpatient

3 Multiple outpatient

4 Multiple inpatient

**3. Have you already been vaccinated against COVID-19?**	1 yes	2 n0

If no:

Why? (Multiple answers possible)	1 My Doctor advised against it.

2 I do not want to be vaccinated.

3 Medical reasons

4 Other reasons: _____________________________________

99 No information

Have you had any preventive (prophylactic)	1 yes

COVID-19 antibody treatments (e.g. Ronapreve®	2 no

Casivirimab/Imdevimab), Xevudy® (Sotrovimab)	99 unknown

Evusheld® (Tixagevimab/Cligavimab)?

4. **How many times have you been vaccinated against COVID-19?**	0 0x

1 1x

2 2x

3 3x

4 4x

5. **When did you receive your first vaccination?**

|__|__|.|__|__|.|__|__|__|__| or  I have not (yet) received my first vaccination.

Day	Month	Year

6. **Which vaccine were you given (1st vaccination)?**

1 AstraZeneca (Vaxzevria®)

2 Biontech/Pfizer (Comirnaty®)

3 Moderna (Spikevax®)

4 Novavax (Nuvaxovid®)

5 Johnson & Johnson

(COVID-19 Vaccine Janssen®)

**7. Did you experience any symptoms after your first vaccination?**

(Multiple answers possible)

0 No symptoms

1 Headache

2 Muscle/joint pain

3 Fever

4 Chills

5 Pain in arm

6 Redness/swelling

7 GvHD occurrence/reactivation

8 Other:_____________________

99 unknown

8. **When did you receive your second vaccination?**

|__|__|.|__|__|.|__|__|__|__| or  I have not (yet) received my second vaccination.

Day	Month	Year

9. **Which vaccine were you given (2nd vaccination)?**

1 AstraZeneca (Vaxzevria®)

2 Biontech/Pfizer (Comirnaty®)

3 Moderna (Spikevax®)

4 Novavax (Nuvaxovid®)

**10. Did you experience any symptoms after your second vaccination?**

(Multiple answers possible)

0 No symptoms

1 Headache

2 Muscle/joint pain

3 Fever

4 Chills

5 Pain in arm

6 Redness/swelling

7 GvHD occurrence/reactivation

8 Other:_____________________

99 unknown

**11. When did you receive your third vaccination?**

|__|__|.|__|__|.|__|__|__|__| or  I have not (yet) received my third vaccination.

Day	Month	Year

**12. Which vaccine were you given (3rd vaccination)?**

1 AstraZeneca (Vaxzevria®)

2 Biontech/Pfizer (Comirnaty®)

3 Moderna (Spikevax®)

4 Novavax (Nuvaxovid®)

**13. Did you experience any symptoms after your third vaccination?**

(Multiple answers possible)

0 No symptoms

1 Headache

2 Muscle/joint pain

3 Fever

4 Chills

5 Pain in arm

6 Redness/swelling

7 GvHD occurrence/reactivation

8 Other:_____________________

99 unknown

**14. When did you receive your fourth vaccination?**

|__|__|.|__|__|.|__|__|__|__| or  I have not (yet) received my fourth vaccination.

Day	Month	Year

**15. Which vaccine were you given (4th vaccination)?**

1 AstraZeneca (Vaxzevria®)

2 Biontech/Pfizer (Comirnaty®)

3 Moderna (Spikevax®)

4 Novavax (Nuvaxovid®)

**16. Did you experience any symptoms after your fourth vaccination?**

(Multiple answers possible)

0 No symptoms

1 Headache

2 Muscle/joint pain

3 Fever

4 Chills

5 Pain in arm

6 Redness/swelling

7 GvHD occurrence/reactivation

8 Other:_____________________

99 unknown
